# Predictors of frequent emergency department visits among hospitalized cancer patients: a comparative cohort study using integrated clinical and administrative data to improve care delivery

**DOI:** 10.1186/s12913-023-09854-1

**Published:** 2023-08-22

**Authors:** Siyana Kurteva, Robyn Tamblyn, Ari N. Meguerditchian

**Affiliations:** 1 Department of Epidemiology and Biostatistics, McGill University, Montreal, Canada; 2 Clinical and Health Informatics Research Group, McGill University, Montreal, Canada; 3 Department of Science, Aetion, Inc, New York, USA; 4 Clinical & Health Informatics Research Group, Department of Medicine, McGill University, 2001 McGill College Avenue, Suite 1200, H3A 1G1 Montreal, Canada; 5 Department of Medicine, McGill University Health Center, Montreal, Canada; 6 McGill University Health Centre, Montreal, Canada; 7 Department of Surgery, McGill University Health Center, Montreal, Canada; 8 Center for Outcomes Research and Evaluation, McGill University Health Centre, Montreal, Canada; 9 St. Mary’s Research Centre, Montreal, Canada

**Keywords:** Emergency department visits, Cancer, Cohort study, Frequent users

## Abstract

**Background:**

Frequent emergency department (FED) visits by cancer patients represent a significant burden to the health system. This study identified determinants of FED in recently hospitalized cancer patients, with a particular focus on opioid use.

**Methods:**

A prospective cohort discharged from surgical/medical units of the McGill University Health Centre was assembled. The outcome was FED use (≥ 4 ED visits) within one year of discharge. Data retrieved from the universal health insurance system was analyzed using Cox Proportional Hazards (PH) model, adopting the Lunn-McNeil approach for competing risk of death.

**Results:**

Of 1253 patients, 14.5% became FED users. FED use was associated with chemotherapy one-year pre-admission (adjusted hazard ratio (aHR) 2.60, 95% CI: 1.80–3.70), ≥1 ED visit in the previous year (aHR: 1.80, 95% CI 1.20–2.80), ≥15 pre-admission ambulatory visits (aHR 1.54, 95% CI 1.06–2.34), previous opioid and benzodiazepine use (aHR: 1.40, 95% CI: 1.10–1.90 and aHR: 1.70, 95% CI: 1.10–2.40), Charlson Comorbidity Index ≥ 3 (aHR: 2.0, 95% CI: 1.2–3.4), diabetes (aHR: 1.60, 95% CI: 1.10–2.20), heart disease (aHR: 1.50, 95% CI: 1.10–2.20) and lung cancer (aHR: 1.70, 95% CI: 1.10–2.40). Surgery (cardiac (aHR: 0.33, 95% CI: 0.16–0.66), gastrointestinal (aHR: 0.34, 95% CI: 0.14–0.82) and thoracic (aHR: 0.45, 95% CI: 0.30–0.67) led to a decreased risk of FED use.

**Conclusions:**

Cancer patients with higher co-morbidity, frequent use of the healthcare system, and opioid use were at increased risk of FED use. High-risk patients should be flagged for preventive intervention.

**Supplementary Information:**

The online version contains supplementary material available at 10.1186/s12913-023-09854-1.

## Introduction

Unplanned acute healthcare use after hospitalization remains an important health system challenge. Emergency department (ED) care account for a large proportion of healthcare system spending [1, 2]. While some ED visits may represent the only option for patients when they experience acute symptoms, the occurrence of a significant number of ED visits, which may lead to negative patient experiences, is medication-related and preventable [3–8]. The impact of ED visits is even more pronounced in cancer patients, as emergency use is associated with treatment errors, delays and adverse outcomes [9–11]. To enhance system performance and design interventions that could accurately identify potential high-risk profiles of vulnerable patients with cancer, modifiable factors that increase the risk of preventable ED visits need to be identified to potentially minimize avoidable morbidity in this vulnerable population [12–14].

Prescription opioid use, an important contributor to adverse outcomes, has increased significantly over the past decade, resulting in a spike in acute healthcare use [15–17]. Pain control is an important cancer care component; cancer patients are at increased risk of opioid-related adverse outcomes, which, in turn, are associated with increased healthcare burden, as up to 40% of them suffer from chronic pain, and may necessiate long-term opioid use [18]. Several studies have estimated the risk of frequent emergency department (FED) use in relationship to patient- and heathcare-related characteristics, and found that young adults, females, having multiple healthcare providers, and chronic conditions were associated with increased risk of FED use [19–22]. However, most of the focus of previous research has been on patient-related factors, and the current evidence is limited to health measures captured in administrative data, which offer incomplete documentation of patients’ health and treatment trajectory. These databases do not have data on hospital-level characteristics or in-hospital medication use. As a result, the potential impact of opioid use, administered in hospital, given as part of a discharge treatment regimen or post-discharge dispensations have received little attention, despite important variations in the risk of acute healthcare services use associated with different patterns and durations of opioid use [22, 23]. Moreover, no study to date has accounted for the risk of competing events such as death [24, 25]. FED use, especially in clinical oncology, represent a complex phenomenon, where patient overall health status and frailty may preclude the occurrence of a subsequent healtchare encounter [26].

The purpose of this study was to identify patents at high risk of FED use post discharge using comprehensive inpatient, outpatient and community-based clinical and population-based administrative databases. A particular focus was placed on the unexplored role of hospital-related determinants and opioid use characteristics.

## Methods

### Study design & setting

The study was performed using the cohort of cancer patients enrolled in a cluster-randomized trial of medication reconciliation conducted at the McGill University Health Centre (MUHC), [27] a > 1000-bed quaternary care teaching hospital in Montreal (Canada) that operates within the universal healthcare plan of the province of Quebec (RAMQ). This RAMQ covers all necessary medical care and includes drug insurance for registrants 65 years of age and older, income security recipients, and those not insured through their employer (approximately 50% of the 8.5 million Quebec population). Ethics approval was provided by the MUHC Ethics Committee Board. Quebec privacy commissioner approval was obtained to link clinical and administrative data from the Quebec Privacy Commissioner. Signed informed consent was provided by all participants. This study adheres to the Strengthening the Reporting of Observational Studies in Epidemiology (STROBE) reporting guideline for observational studies [28].

### Participants

A prospective cohort of cancer patients with non-metastatic disease (stage I-III) admitted to medical and surgical units between October 2014 and November 2016 was followed one-year post-discharge. Patients had to be 18 years of age or older at admission, admitted from the community or transferred from another hospital or ER, with at least one-year continuous provincial healthcare coverage prior to admission. Furthermore, patients had to be discharged alive and have an active confirmed diagnosis of cancer (ICD9:1400–1999; ICD10: C77-C80, C18-20, C33, C45, C61), in the 12 months prior to or at admission. To assess the associations between initial opioid prescription characteristics, patients had to fill at least one opioid prescription post-discharge before their first occurrence of a post-discharge ED encounter.

### Data sources

Multiple data sources were assembled and linked to address the study objectives. For each patient, admission and discharge dates, discharge unit, patient demographics, active co-morbidities, and interventions received during hospitalization were retrieved from the hospital discharge abstract database. Community-based, ER and hospital-based medical services, and medication use in the year prior to and after the hospitalization were retrieved from RAMQ’s medical and pharmacy administrative claims data. In-hospital medications as well as those prescribed at discharge were retrieved from the MUHC drug information system. Combining these data sources provided a complete record of both community-based and in-hospital medication use. Additional information pertaining to the hospital stay was abstracted from the medical chart. Medical conditions and comorbidities were coded from administrative claims using the International Classification of Disease 9th revision (ICD9 codes) and medications were classified according to the Anatomical Therapeutic Chemical Classification System code (ATC). Types of surgery were coded and classified using the Canadian Classification of Health Interventions (CCI).

### FED use and predictors

#### Emergency department use

The main outcome was FED use (≥ 4 ED visits) in the year following hospital discharge and it was ascertained using the provincial RAMQ medical services claims databases. This definition for FED use was based on the most widely used definition found in the available literature and was deemed to represent a valid threshold [29–32].

#### Patient-related characteristics

For patient-related factors, RAMQ drug programs and hospital charts were used to collect information on age at admission and sex, as these characteristics were shown to be associated with FED visits in previous studies [22, 25, 33, 34]. Patients’ income-indexed copayment plan for medications was used as a proxy for socioeconomic status since employment status, and higher income have been associated with lower rates of FED use in previous studies [19, 34, 35]. Under Quebec’s public drug insurance plan, patients could fall into one of three income-based copayment plans: 1) no copayment (free medications), 2) partial copayment (25% per prescription up to a maximum of $600 annually), or 3) maximum copayment (25% per prescription up to a maximum of $1,000 annually). Baseline comorbidities were measured using the Charlson Comorbidity Index (CCI). Specific comorbidities, with known associations were also investigated. These included targeted psycho-social (history of mental health diagnoses, substance and/or alcohol abuse/dependence), and physical comorbidities (cardiovascular disease, renal disease, respiratory diseases, pneumonia, chronic obstructive pulmonary disease, chronic pain). Type of cancer diagnosis (tumor site) in the year prior to admission and updated during the hospital stay (see Supplementary Material Appendix A for a full list of covariates included in the model).

#### Healthcare utilization

Using RAMQ medical services databases in the one year prior to admission, we collected information on the receipt of radiotherapy and/or chemotherapy services, number of outpatient visits, number of prescribing physicians, dispensing pharmacies, and the occurrence of a hospitalization or ED visit in the past year.

#### Medication use and hospital characteristics

Pre-admission use of opioids, benzodiazepine, non-opioid analgesics and antidepressant were measured using the RAMQ prescription claims database in the 12 months prior to admission as well as during the index hospitalization using data from the hospital pharmacy system. In-hospital characteristics including the reason for the index admission, type of surgery received, length of hospital stay, admission to the intensive care unit (ICU), discharge destination were retrieved from the MED-ECHO hospitalization database and the use of electronic medication reconciliation software to finalize the discharge prescription.

#### Prescribing physician characteristics

Uniquely to this study, we retrieved organizational and physician’s characteristics from the hospital data warehouse including physician’s years in practice, status (resident versus attending physician).

#### Opioid discharge prescription and initial opioid dispensation characteristics

Treatment changes made to opioid medications from the community were evaluated by using patients’ discharge prescriptions data in comparison to their community drug list. These methods were previously described elsewhere [15, 36]. Briefly, a categorical variable for whether a given opioid was stopped, continued or newly prescribed was derived. A binary variable for the presence of an opioid as part of the discharge pain regimen was constructed: patients with newly added or continued opioids were flagged as having an opioid prescription. The first post-discharge opioid molecule (e.g., oxycodone, hydromorphone), dose (converted to morphine milligram equivalents (MME) formulation (short-acting vs long-acting), duration, and number of opioid prescriptions dispensed on the same day were measured using RAMQ pharmacy claims (Supplementary Material, Appendix B-C).

### Statistical analyses

Descriptive statistics were used to characterize cancer patients who became FED users compared to those who did not [37]. Multivariable Cox Proportional Hazards (PH) models were utilized to determine the association between patient-, drug- and system-level characteristics and FED use. Start of follow-up corresponded to the discharge date from the index hospitalization. End of follow-up corresponded to the day when the patient first met the definition of FED user, death, or to the end of the 12 month follow-up, whichever occurred first. The Lunn-McNeil (LM) approach for competing risk analyses extended to Cox PH models was used to account for the possibility of death during the follow-up period [38–40]. Event indicator variables were created for the corresponding event types (either FED use or death). The LM method uses a data augmentation approach to estimate a single completing risk model, which stratifies the results by the distinct baseline hazards for each event type [40, 41]. Final variables included in the model were based on backward selection with a p-value of 0.1 used for a variable inclusion with the exception of patient age and sex that were included to minimize residual confounding. The “events-per-variable” rule was used to guide the final number of variables in the model (excluding characteristics of the initial opioid dispensation), with an event-to-variable ratio of 10 [42]. For each covariate, the results were presented as adjusted hazard ratios (aHR), with 95% confidence intervals (CI). We tested the PH assumption, both globally and for each covariate in the multivariable Cox model, using the Grambsch and Therneau approach [43]. For covariates for which the PH hypothesis was violated (p < 0.05), we relied on smooth residual plots to assess how the corresponding adjusted hazard ratio varied over the follow-up (Supplementary Material Appendix D). Additionally, we also examined the reasons for the ED visits, as documented in the medical claims databases, to provide additional information about the type of visits the frequent and non-frequent ED users experienced during the follow-up period (Appendix E).

To investigate the association between characteristics of the initial opioid dispensation and FED use, we ran a separate Cox PH model accounting for all variables, including the opioid-related factors in the backward selection, but only among patients who filled at last one opioid prescription post-discharge. This additional eligibility restriction was necessary in order to avoid immortal time-bias as well as to reduce confounding by disease severity by only comparing these characteristics among opioid users [44, 45]. To maintain an appropriate temporal sequence that properly places the exposure relative to when the outcome occurs, only patients who filled their first post-discharge opioid prescription before they had their first post-discharge ED encounter were included in the model [46]. Cohort entry for this model was time of first dispensation to further reduce the opportunity for immortal time bias [47].

All analyses were performed using SAS version 9.4 (SAS Institute, Cary, NC) and statistical models were analyzed using R (R Core Team, 2017).

## Results

**Fig. 1:**
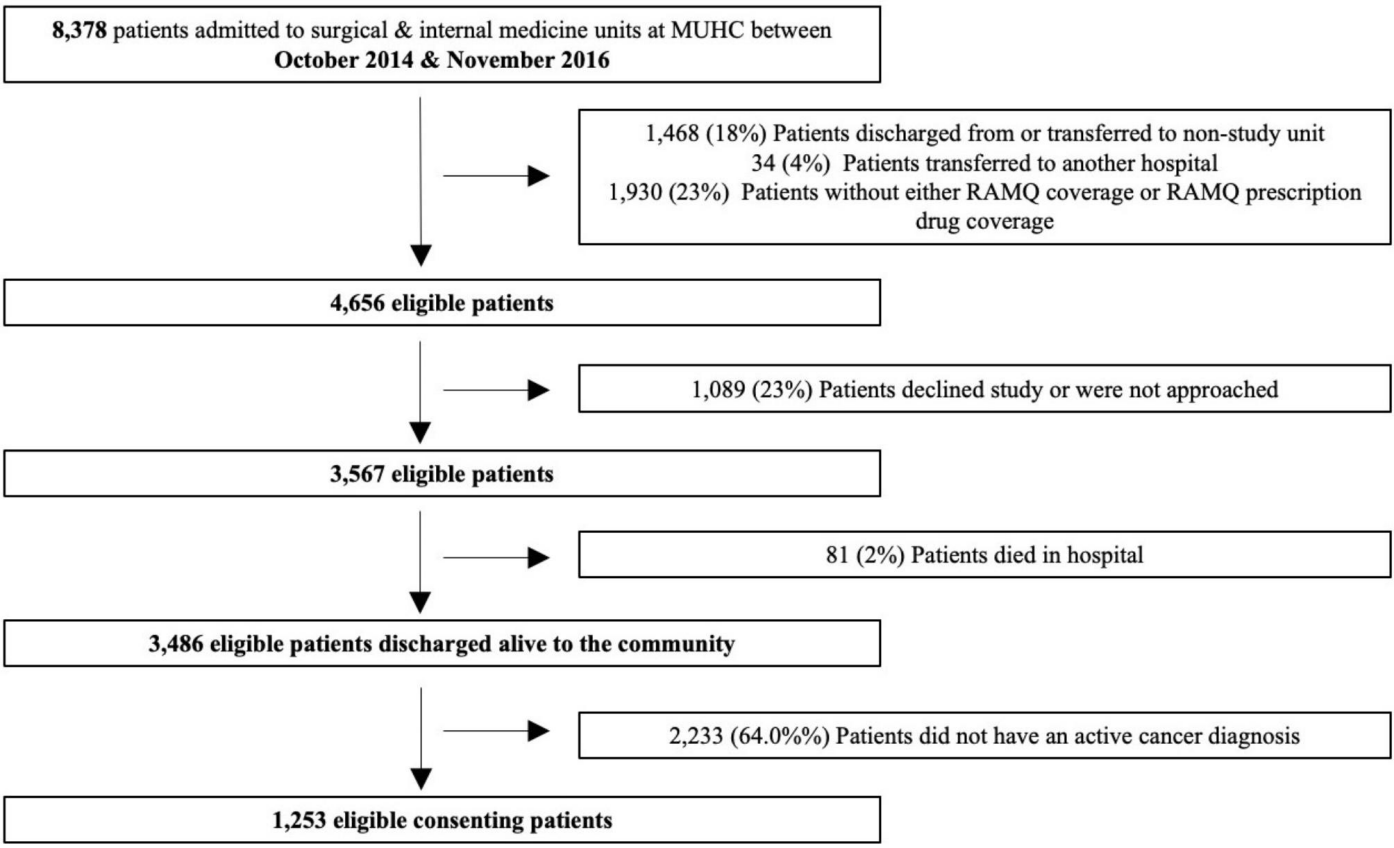
Flowchart of eligible cancer patients

A total of 1253 cancer patients (Fig. 1) were discharged during the study period, with a mean age of 70.9 years. Of these, 57.5% were male, and 41.8% were discharged from a medical service (Table 1). The most frequent cancers were respiratory (38.9%) and upper digestive (24.7%) (Table 1).

**Table 1:** Baseline characteristics of cancer patients according to their frequency of emergency department use

	Overall n = 1253	Non-Frequent Users (0–3 ED visits) n = 1071 (85.5%)	Frequent Users (≥ 4 ED visits) n = 182 (14.5%)
**Patient-related characteristics**			
**Age at admission (years)**			
Mean age (SD)	70.9 (11.8)	71.3 (11.8)	69.1 (11.5)
≤64	300 (23.9)	250 (23.3)	50 (27.5)
>64	953 (76.1)	821 (76.7)	132 (72.5)
	***N (%)***	***N (%)***	***N (%)***
**Gender**			
Female	532 (42.5)	459 (42.9)	73 (40.1)
Male	721 (57.5)	612 (57.1)	109 (59.9)
**Medication copayment plan**			
No copayment	196 (15.6)	161 (15.0)	35 (19.2)
Partial copayment	294 (23.5)	248 (23.2)	46 (25.3)
Maximum copayment	763 (60.9)	662 (61.8)	101 (55.5)
**Healthcare use in the one year pre-admission period**			
	***Mean (SD)***	***Mean (SD)***	***Mean (SD)***
Emergency department (ED) visits	9.2 (14.5)	8.5 (14.3)	13.0 (15.5)
Hospitalizations	0.93 (2.21)	0.89 (2.3)	1.1 (1.8)
Ambulatory visits	14.5 (12.1)	14.1 (12.2)	16.9 (10.9)
	***N (%)***	***N (%)***	***N (%)***
Radiotherapy	192 (15.3)	138 (12.9)	54 (29.7)
Chemotherapy	239 (19.1)	168 (15.7)	71 (39.0)
	***Mean (SD)***	***Mean (SD)***	***Mean (SD)***
Number of prescribing physicians	4.8 (3.4)	4.6 (3.2)	5.9 (3.9)
Number of dispensing pharmacies	1.4 (0.85)	1.4 (0.86)	1.5 (0.81)
Active prescriptions at admission	8.7 (9.7)	8.5 (9.5)	10.2 (11.1)
**Medication use in the one year pre-admission period**			
	***N (%)***	***N (%)***	***N (%)***
Active opioid prescription at admission	207 (16.5)	159 (14.9)	48 (26.4)
History of opioid use	505 (40.3)	407 (38.0)	98 (53.9)
History of > 3 opioid dispensations	45 (3.6)	37 (3.5)	8 (4.4)
History of analgesics use	505 (40.3)	413 (38.6)	92 (50.6)
History of antidepressant use	270 (21.6)	226 (21.1)	44 (24.2)
History of benzodiazepines	503 (40.1)	403 (37.6)	100 (54.9)
**Targeted comorbidities that may increase the risk of hospitalizations/ED visits**			
**Charlson Comorbidity Index**			
0	14 (1.1)	14 (1.3)	0 (0)
1–2	247 (19.7)	232 (21.7)	15 (8.2)
≥3	992 (79.2)	825 (77.0)	167 (91.8)
Cardiovascular Diseases	602 (48.0)	508 (47.4)	94 (51.7)
Cerebrovascular Diseases	114 (9.1)	92 (8.6)	22 (12.1)
Pneumonia	124 (9.9)	101 (9.4)	23 (12.6)
Chronic obstructive pulmonary disease	349 (27.9)	292 (27.3)	57 (31.3)
Diabetes	245 (19.6)	197 (18.4)	48 (26.4)
Renal Disease	116 (9.3)	103 (9.6)	13 (7.1)
History of mental illness	190 (15.2)	158 (14.8)	32 (17.6)
History of substance& alcohol abuse	37 (2.9)	30 (2.8)	7 (3.9)
History of pain syndromes	515 (41.1)	431 (40.2)	84 (46.2)
**Cancer diagnoses**			
Digestive	309 (24.7)	257 (24.0)	52 (28.6)
Lung	488 (38.9)	408 (38.1)	80 (43.9)
Breast cancer	274 (21.9)	232 (21.7)	42 (23.1)
Urologic	248 (19.8)	214 (19.9)	34 (18.7)
Unspecified Cancer	88 (7.0)	76 (7.1)	12 (6.6)
**In-hospital medication use**			
Antidepressants	220 (17.6)	184 (17.2)	36 (19.8)
Opioids	968 (77.3)	826 (77.1)	142 (78.0)
Benzodiazepines	855 (68.2)	731 (68.3)	124 (68.1)
Analgesics	1163 (92.8)	993 (92.7)	170 (93.4)
**Characteristics of the index hospitalization**			
**Reasons for admission**			
Cancer	368 (29.5)	315 (29.5)	53 (29.3)
Respiratory	201 (16.1)	180 (16.9)	21 (11.6)
Cardiovascular	177 (14.2)	160 (15.0)	17 (9.4)
Digestive	80 (6.4)	66 (6.2)	14 (7.7)
Genitourinary	60 (4.8)	55 (5.2)	5 (2.8)
Health services (examinations)	43 (3.5)	8 (4.4)	35 (3.3)
Infections	36 (2.9)	23 (2.2)	13 (7.2)
Musculoskeletal	29 (2.3)	25 (2.3)	4 (2.2)
Injection poisonings	20 (1.6)	18 (1.7)	2 (1.1)
Dermatological	20 (1.6)	16 (1.5)	4 (2.2)
Immune system	17 (1.4)	14 (1.3)	3 (1.7)
Metabolic	16 (1.3)	9 (0.8)	7 (3.9)
Other ^1^	165 (13.2)	137 (12.8)	28 (15.4)
Neurological/behavioral	11 (0.9)	10 (9.3)	1 (5.5)
**Received surgery**	639 (51.0)	563 (52.6)	76 (41.7)
* Type of Surgery Received*			
Cardiac	118 (9.4)	108 (10.1)	10 (5.5)
Gastrointestinal	49 (3.9)	44 (4.1)	5 (2.8)
Thoracic	417 (33.3)	358 (33.4)	59 (32.4)
Unrelated	55 (4.4)	53 (4.9)	2 (1.1)
**Length of hospital stay, days**			
Mean (SD)	8.9 (11.6)	8.7 (11.7)	8.6 (11.4)
<6	166 (13.3)	143 (13.4)	23 (12.6)
≥6	1087 (86.8)	928 (86.6)	159 (87.4)
Admission to the ICU	97 (7.7)	81 (7.7)	16 (8.8)
Medication reconciliation used	291 (23.2)	255 (23.8)	36 (19.8)
**Attending physician years of practice**			
1–20	350 (27.9)	291 (27.2)	59 (32.4)
20–40	661 (52.8)	575 (53.7)	86 (47.3)
>40	242 919.3)	205 (19.1)	37 (20.3)
**Discharge prescription signed by**			
Attending physician	280 (22.3)	241 (22.5)	39 (21.4)
Resident	973 (77.6)	830 (77.5)	143 (78.6)
**Home discharge destination**			
Home	1147 (91.5)	975 (91.0)	172 (94.5)
Long-term care facility	106 (8.5)	96 (8.9)	10 (5.5)
**Pain regimen at discharge**			
Opioid prescription	1017 (81.2)	870 (81.2)	147 (80.8)
Analgesic prescription	814 (64.9)	694 (64.8)	120 (65.9)
**Characteristics of the initial opioid dispensation post-discharge** ^**2,3**^			
Filled an opioid prescription post-discharge	475 (37.9)	357 (33.3)	118 (64.8)
Filled an opioid prescription within 30 days’ post-discharge	444 (93.5)	331 (92.7)	113 (95.8)
Filled ≥1 type of opioid	36 (7.6)	26 (7.3)	10 (8.5)
**MME Dose**			
≤20	100 (21.1)	74 (20.7)	26 (22.0)
20–50	267 (56.2)	201 (56.3)	66 (55.9)
50–90	93 (19.6)	73 (20.5)	20 (16.9)
>90	15 (3.2)	9 (2.5)	6 (5.1)
**Days’ Supply**			
≤7	238 (50.1)	181 (50.7)	57 (48.3)
>7	237 (49.9)	176 (49.3)	61 (51.7)
**Formulation**			
Short-acting	444 (93.5)	335 (93.8)	109 (92.4)
Long-acting	31 (6.5)	22 (6.2)	9 (7.6)
**Type of opioid**			
Codeine	10 (2.1)	9 (1.9)	1(0.9)
Hydromorphone	151 (31.8)	104 (29.1)	47 (39.8)
Oxycodone	272 (57.3)	215 (60.2)	57 (48.3)
Fentanyl	9 (1.9)	8 (2.2)	1 (0.9)
Methadone	2 (0.4)	1 (0.3)	1 (0.9)

ED = emergency department; SD = standard deviation; ICU = intensive care unit; MME = milligram morphine equivalent

^1^ Other: pain, vomiting, nausea, dizziness, swelling

^2^ These numbers are presented only among those who filled an opioid dispensation post-discharge, before their first

ED visit following the index hospitalization (n = 792)

^3^Percentage of opioid characteristics is calculated based on the total number of patients with at least one dispensation (n = 475)

Overall, 14.5% (n = 182) of patients became FED users and 19.3% (n = 242) died during the follow-up (Table 1). The mean time of follow-up for FED users was 208.1 days. When compared to patients with < 4 ED visits, patients who became FED users were more likely to have had a higher mean number of ED (13.0 vs 8.5) and ambulatory visits (16.9 vs 14.1) in the pre-admission year, had received radiotherapy (29.7% vs 12.9%) or chemotherapy during those 12-months (39.0% vs 15.7%), and already had an active opioid prescription at admission (26.4% vs 13.0%). FED users were also more likely to have had a history of opioid (53.9% vs 38.0%), analgesic (50.6% vs 38.6%) and benzodiazepine (54.9% vs 37.6%) use before their admission, as well as have a higher (≥3) CCI index (91.8% vs 77.0%). FED users were less likely to have received surgery during their index hospitalization (41.7% vs 52.6%).

**Fig. 2:**
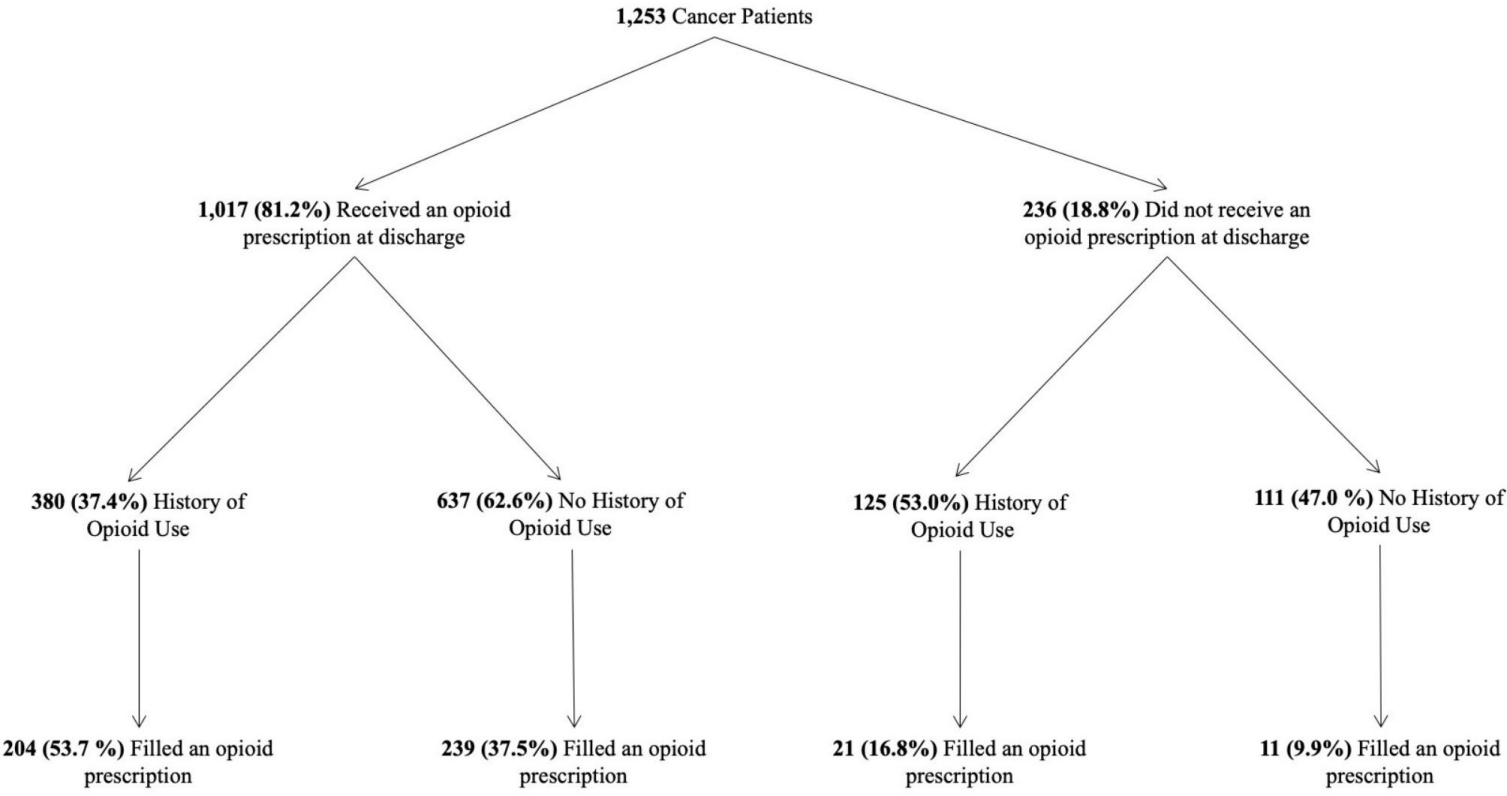
Flowchart of opioid consumption according to cancer patients’ receipt of opioid prescription at discharge, previous history of opioid use and post-discharge dispensations

At discharge, 81.2% of patients received an opioid prescription (Fig. 2, Table 2). Most discharge opioid prescriptions were newly initiated, as 62.6% of patients did not have an opioid dispensation in the year proceeding their hospitalization (Fig. 2). Once in the community, 37.9% of patients had at least one opioid dispensed, which always preceded their ED visit (Table 1).

**Table 2:** Association between frequent emergency department use and patient-, medication-, and system-related characteristics (n = 1253)

Variable	Adjusted HR^1^ (95% CI)
**Demographics**	
Age	
<65	Reference
≥65	0.9 (0.7–1.2)
Gender	
Female	Reference
Male	1.2 (0.9–1.6)
**Healthcare use characteristics**	
Emergency department (ED) visits	
0	Reference
≥1	**1.8 (1.2–2.8)**
Ambulatory visits	
<15	Reference
≥15	1.2 (0.89–1.7)
Radiotherapy	
No	Reference
Yes	1.4 ( 0.91 − 2.0)
Chemotherapy	
No	Reference
Yes	**2.6 (1.8–3.7)**
**Comorbidities**	
Charlson Comorbidity Index	
0–2	Reference
≥3	**2.0 (1.2–3.4)**
Renal disease	
No	Reference
Yes	0.6 (0.4–1.1)
Diabetes	
No	Reference
Yes	**1.6 (1.1–2.2)**
Heart disease	
No	Reference
Yes	**1.5 (1.1–2.2)**
Lung cancer	
No	Reference
Yes	**1.7 (1.1–2.4)**
**Medication use**	
History of benzodiazepine use	
No	Reference
Yes	**1.7 (1.1–2.4)**
History of opioid use	
No	Reference
Yes	**1.4 (1.0–1.9)**
**In-hospital characteristics**	
* Type of surgery*	
No surgery	Reference
Cardiac	**0.33 (0.16–0.66)**
Gastrointestinal	**0.34 (0.14–0.82)**
Thoracic	**0.45 (0.30–0.67)**
Unrelated	**0.21 (0.05–0.89)**
Admission to the ICU	
No	Reference
Yes	1.67 (0.98–2.8)
Hospital LOS, days	
<6	Reference
≥6	1.2 (0.76–1.8)
Discharge prescription for a non-opioid analgesic	
No	Reference
Yes	1.32 (0.95–1.9)

HR = hazard ratio, CI = confidence interval, LOS = length of hospital stay

^1^Adjusted for all the variables in the table. Covariate selection was based on backward selection, using a p-value of 0.1 for variable inclusion, as well as a number of characteristics with low p-values. The final number of variables selection was based on the events-per-variable rule, where the total number of variables in the final model did not fall beyond an event-to-variable ratio of 10. Statistically significant findings are bolded. In total, there were 242 patients (19.3%) who had died before having the opportunity to become frequent emergency department users.

The most common dispensed opioids were oxycodone (57.3%) and hydromorphone (31.8%) (Table 1). With respect to post-discharge opioid consumption, FED users had similar characteristics to non-FED users in terms of the dose, duration and formulation of the first post-discharge opioid prescription but were more likely to have filled a prescription for hydromorphone (39.8% vs 29.1%) (Table 1).

In the multivariable Cox model (Table 2), patients who had a history of ≥ 1 ED visit pre-admission were associated with an increased likelihood of transitioning into a FED user post-discharge (aHR 1.80, 95% CI 1.20–2.80). Patients receiving chemotherapy preadmission were 2.6 times more likely to become FED users (aHR 2.60, 95% CI, 1.80–3.70). Having a higher CCI (≥3) (aHR: 2.0, 95% CI: 1.2–3.4), a history of diabetes (aHR: 1.60, 95% CI: 1.10–2.20), heart disease (aHR: 1.50, 95% CI: 1.10–2.20), or lung cancer (aHR: 1.70, 95% CI: 1.10–2.40), were all independently associated with an increased likelihood of becoming a FED user. Having had surgery (cardiac (aHR: 0.33, 95% CI: 0.16–0.66), gastrointestinal (aHR: 0.34, 95% CI: 0.14–0.82) and thoracic (aHR: 0.45, 95% CI: 0.30–0.67) led to a decreased risk of FED use. Patients with a preadmission history of opioid use had a 40% higher risk of becoming FED users during the one-year post-discharge (aHR 1.40, 95% CI 1.10–1.90) and a 70% increased risk of FED use with a history of benzodiazepine use (aHR 1.70, 95% CI 1.10–2.20). In the subset of 475 (37.9%) of patients who filled at least one opioid dispensation post discharge, having had ≥15 ambulatory visits (aHR 1.54 95% CI 1.06–2.34) preadmission was also associated with FED use (as compared to the main model) (Table 3)

**Table 3:** Association between frequent emergency department use and patient-, medication-, system- and opioid-related characteristics among patients who filled an opioid prescription before their first post-discharge emergency department visit (n = 475)

Variable	Adjusted HR^1^ (95% CI)
**Healthcare use characteristics**	
Emergency department (ED) visits	
0	Reference
≥1	**1.73 (1.08–2.75)**
Ambulatory visits	
<15	Reference
≥15	**1.54 (1.06–2.34)**
Chemotherapy	
No	Reference
Yes	**2.05 (1.40–3.00)**
**Comorbidities**	
Renal disease	
No	Reference
Yes	0.46 (0.20–1.26)
Heart disease	
No	Reference
Yes	**1.95 (1.27–2.97)**
Diabetes	
No	Reference
Yes	**1.75 (1.14–2.69)**
Lung cancer	
No	Reference
Yes	**1.71 (1.07–2.74)**
**Medication use**	
Active opioid prescription at admission	
No	Ref
Yes	0.83 (0.52–1.32)
**In-hospital characteristics**	
Antidepressant use	
No	Ref
Yes	1.19 (0.76–1.87)
*Type of surgery*	
No surgery	Reference
Cardiac	**0.18 (0.06–0.49)**
Gastrointestinal	**0.35 (0.14–0.93)**
Thoracic	**0.42 (0.26–0.70)**
Unrelated	**0.18 (0.04–0.80)**
Admission to the ICU	
No	Reference
Yes	2.20 (0.99–4.88)
Discharge prescription for an opioid medication	
No	Reference
Yes	1.53 (0.95–2.47)

HR = hazard ratio, CI = confidence interval, LOS = length of hospital stay

^1^Adjusted for all the variables in the table. Covariate selection was based on backward selection, using a p-value of 0.1 for variable inclusion, as well as a number of characteristics with low p-values. The final number of variables selection was based on the events-per-variable rule, where the total number of variables in the final model did not fall beyond an event-to-variable ratio of 10. Statistically significant findings are bolded. In total, there were 92 patients (19.4%) who had died before having the opportunity to become frequent emergency department users.

The PH hypothesis was rejected by the global test (p = 0.021 and for three of the selected covariates (p < 0.05 for each). Appendix D of the Supplementary Material describes how the corresponding aHR’s of each of these covariates varied with increasing follow-up.

With respect to the reasons for the ED visits, in Appendix E, we see the descriptive breakdown comparing the most common documented reasons in the ED visits experienced by non-frequent ED users and the FED users. In the breakdown of the reasons associated with the ED visits (multiple reasons could be documented within the same healthcare encounter), among the FED users, 50% of the visits were for cancer-related diagnoses, as compared to 36.6% for the non-frequent users. We also observed higher percentage of documented reasons for infections and cardiovascular related symptoms. The percentage of visits related to the receipt of clinical follow-up care within the ED setting was similar across non-frequent ED users and FED users.

## Discussion

We found that cancer patients recently discharged from hospital are at risk of FED visits. Higher pre-admission ED use, ambulatory visits, receipt of radiotherapy and/or chemotherapy, a greater number of comorbidities, a history of heart disease, diabetes, or lung cancer, and pre-admission use of opioids and benzodiazepines were associated with a higher risk of FED use.

Several studies have shown a link between FED use and higher number of past ED visits, history of heart disease, and increased comorbidity profile [22, 25, 33, 35, 48–50]. In this study, however, we were able to investigate the potential impact of additional system-level, organizational or opioid related medication factors on FED use [29–31, 51], which led to interesting observations. For example, we found that the receipt of any type of surgery, was associated with a decreased risk of FED use. This finding might be related to the implementation of standardized care pathways that include discharge planning and better follow-up protocols [52, 53]. However, this finding may be a reflection of having patients in the study cohort that are at an early stage of their cancer disease and, thus, excluding the sickest population; the results should be replicated, in addition to investigating the effect measure modification of having surgery on the risk of FED, using larger cohorts within existent databases that link clinical information to administrative databases.

Previous studies have found opioid dose to be associated with increased risk of long-term opioid use and its associated adverse events [54–56]. In our study, we found that opioid prescriptions often went unfilled and opioid use was not associated with increased risk of FED, which may be a reflection of patients included at an early stage of their cancer disease. While our findings showed that initial doses of dispensed opioids did not exceed recommended thresholds and did not lead to increased risk of incurring a high-number of acute healthcare events [57], a longer follow-up window may be needed to accurately reflect the role of opioid patterns of use and dose on the risk of FED use. However, over time, patients, progressing to later disease stages, may add several medications to maximize pain control, accumulating longer use over time [58–60], which could consequently result in increased risk of opioid-related morbidity and healthcare utilization.

Challenges in cancer-related pain management have highlighted the need to link electronic health records with prescription drug monitoring to better track patients data and provide safe transitions of care [61]. In out sudy, we demonstrated that it is possible to link these types of data sources, which, in turn, allowed us to accurately track all in-patient care as well as discharge prescriptions to identify at-risk profiles. Thus, this approach offers the possibility of developing real-time tools that could flag medications’ status at transitions in care, as well as document any complexities in medication regimens that may require further monitoring, and help clinicians to re-evaluate high-risk cancer patients.

The study’s strengths included comprehensive linked data on each patient’s care trajectory including detailed clinical data, information on opioid prescription written at the transitions in care, as well as administrative data that enabled us to measure patient attributes that would not normally be feasible. Accessing information from a single, universal health insurance coverage provider further strengthens the findings, by detailing consumption of all healthcare, without regards to type or site. Risk of competing events, a concept first introduced within clinical research in the oncology field [62], and particularly important in cancer patients, who are at a higher risk of death, has not been previously explored when assessing predictors of FED use. To our knowledge, this study is the first to investigate patient, system-level and opioid-related characteristics, and provide further insights into the opioid patterns of use that may lead to increased healthcare use using adequate modelling techniques accounting for competing risk of death.

Some limitations of our work merit emphasis. First, this was an observational study and, although we used robust statistical procedures, the results should be interpreted cautiously without inferring causality. We sought to minimize confounding of FED use by severity by including a wealth of detailed information that reflect patient’s disease severity and medication use. Nevertheless, despite considering the receipt of chemotherapy and radiotherapy, there could still be a possibility for residual confounding by cancer severity. Second, results may not be generalizable to other settings. Third, we used a definition of ≥ 4 ED visits to classify frequent users and, since other studies have used thresholds ranging from 4 to 12 visits per year, our results may depend on how frequent use is defined. Fourth, while we presented the documented reasons for the ED visits, we did not study the independent associations with outcome-specific ED visits; we also did not include information on the availability of follow-up care receipt, acknowledging the possibility that some of the ED visits may be truly emergent and necessary given the patient disease profile. Fifth, as part of the prescribing characteristics, we did not look at whether the prescribing physicians were specialists, generalists, or palliative care physicians, and as such, we cannot comment on the role of the type of prescriber in the studied associations; this area should be explored in future avenues of research. Finally, generalizability of our findings may be limited due to studying only stage I-III patients, which excludes the sickest of the population, and represents patients that are at the earliest stage of their cancer progression.

In conclusion, this study used comprehensive data to assess the association between various risk factors with FED use. These results may help hospitals and community health providers to improve the quality of cancer care through providing alternative treatments for high-risk cancer patients.

### Electronic Supplementary Material

Below is the link to the electronic supplementary material

Supplementary Material 1

## Data Availability

Our Research Ethics Board (REB) approval for this project limits data access to Robyn Tamblyn’s team. To provide access to other researchers, they would need to obtain permission from the Institut de la Statistique du Québec for their project that would use governmental provincial administrative (RAMQ) data. More details regarding eligibility to request data can be found here: https://statistique.quebec.ca/research/#/a-propos/utilisation-guichet. To use the clinical data, they would have to apply for REB approval for the project, and request that a de-identified copy of the data be made available as consent was provided by patients for only Robyn Tamblyn. More information regarding access to data can be found here: https://statistique.quebec.ca/research/#/demarche/etape-par-etape. A link to submit a data request can be found here: https://statistique.quebec.ca/zone-chercheur/#/login.

## References

[1] Finlayson K, Chang AM, Courtney MD (2018). Transitional care interventions reduce unplanned hospital readmissions in high-risk older adults. BMC Health Serv Res.

[2] Leonardsen A-CL, Grøndahl VA, Ghanima W (2017). Evaluating patient experiences in decentralised acute care using the Picker Patient Experience Questionnaire; methodological and clinical findings. BMC health services research 2017/09/29.

[3] Hudon C, Chouinard MC, Diadiou F, Lambert M, Bouliane D (2015). Case Management in Primary Care for Frequent Users of Health Care Services With Chronic Diseases: A Qualitative Study of Patient and Family Experience. *Annals of family medicine*. Nov.

[4] Hudon C, Chouinard MC, Dubois MF et al. Case Management in Primary Care for Frequent Users of Health Care Services: A Mixed Methods Study. *Annals of family medicine*. May 2018;16(3):232–9. 10.1370/afm.2233. 10.1370/afm.2233 PMC5951252 29760027

[5] Sargent P, Pickard S, Sheaff R, Boaden R (2007). Patient and carer perceptions of case management for long-term conditions. Health & social care in the community Nov.

[6] Williams V, Smith A, Chapman L, Oliver D (2011). Community matrons–an exploratory study of patients’ views and experiences. J Adv Nurs Jan.

[7] Brooks GA, Abrams TA, Meyerhardt JA (2014). Identification of potentially avoidable hospitalizations in patients with GI cancer. J Clin Oncol Feb.

[8] Vandyk AD, Harrison MB, Macartney G, Ross-White A, Stacey D (2012). Emergency department visits for symptoms experienced by oncology patients: a systematic review. Support Care Cancer Aug.

[9] Bese NS, Hendry J, Jeremic B (2007). Effects of prolongation of overall treatment time due to unplanned interruptions during radiotherapy of different tumor sites and practical methods for compensation. Int J Radiat Oncol Biol Phys Jul.

[10] Gallaway MS, Idaikkadar N, Tai E (2021). Emergency department visits among people with cancer: Frequency, symptoms, and characteristics. J Am Coll Emerg Physicians Open Jun.

[11] Mayer DK, Travers D, Wyss A, Leak A, Waller A (2011). Why do patients with cancer visit emergency departments? Results of a 2008 population study in North Carolina. J Clin Oncol Jul.

[12] Moe J, Kirkland SW, Rawe E (2017). Effectiveness of Interventions to Decrease Emergency Department Visits by Adult Frequent Users: A Systematic Review. Acad Emerg medicine: official J Soc Acad Emerg Med Jan.

[13] Mitchell MS, Leon CLK, Byrne TH, Lin WC, Bharel M (2017). Cost of health care utilization among homeless frequent emergency department users. Psychol Serv May.

[14] McConville S, Raven MC, Sabbagh SH, Hsia RY. Frequent Emergency Department Users: A Statewide Comparison Before And After Affordable Care Act Implementation. *Health affairs (Project Hope)*. Jun 2018;37(6):881–9. 10.1377/hlthaff.2017.0784. 10.1377/hlthaff.2017.0784 29863931

[15] Kurteva S, Abrahamowicz M, Gomes T, Tamblyn R (2021). Association of Opioid Consumption Profiles After Hospitalization With Risk of Adverse Health Care Events. JAMA Netw Open May.

[16] Miller M, Barber CW, Leatherman S (2015). Prescription opioid duration of action and the risk of unintentional overdose among patients receiving opioid therapy. JAMA Intern Med Apr.

[17] Saunders KW, Dunn KM, Merrill JO, et al. Relationship of opioid use and dosage levels to fractures in older chronic pain patients. J Gen Intern Med. Apr 2010;25(4):310–5. 10.1007/s11606-009-1218-z. 10.1007/s11606-009-1218-z PMC2842546 20049546

[18] Paice JA. Navigating Cancer Pain Management in the Midst of the Opioid Epidemic. Oncology (Williston Park, NY). Aug 15 2018;32(8):386 – 90, 403. 30153316

[19] Galvin R, Gilleit Y, Wallace E (2017). Adverse outcomes in older adults attending emergency departments: a systematic review and meta-analysis of the Identification of Seniors At Risk (ISAR) screening tool. Age and ageing Mar.

[20] Lash RS, Bell JF, Reed SC (2017). A Systematic Review of Emergency Department Use Among Cancer Patients. *Cancer nursing*. Mar/Apr.

[21] Moriya AS, Miller GE. Any Use and Frequent Use of Opioids among Elderly Adults in 2015–2016, by Socioeconomic Characteristics. Statistical Brief (Medical Expenditure Panel Survey (US)). Agency for Healthcare Research and Quality (US); 2001. 30395428

[22] Scott J, Strickland AP, Warner K, Dawson P (2014). Frequent callers to and users of emergency medical systems: a systematic review. Emerg Med journal: EMJ Aug.

[23] Kanzaria HK, Niedzwiecki M, Cawley CL et al. Frequent Emergency Department Users: Focusing Solely On Medical Utilization Misses The Whole Person. *Health affairs (Project Hope)*. Nov 2019;38(11):1866–1875. 10.1377/hlthaff.2019.00082. 10.1377/hlthaff.2019.00082 31682499

[24] Chiu Y, Racine-Hemmings F, Dufour I (2019). Statistical tools used for analyses of frequent users of emergency department: a scoping review. BMJ Open.

[25] Burns TR (2017). Contributing factors of frequent use of the emergency department: A synthesis. Int Emerg Nurs Nov.

[26] Tan KS, Eguchi T, Adusumilli PS (2018). Competing risks and cancer-specific mortality: why it matters. Oncotarget Jan.

[27] Tamblyn R, Abrahamowicz M, Buckeridge DL (2019). Effect of an Electronic Medication Reconciliation Intervention on Adverse Drug Events: A Cluster Randomized Trial. JAMA Netw Open.

[28] Vandenbroucke JP, von Elm E, Altman DG (2007). Strengthening the Reporting of Observational Studies in Epidemiology (STROBE): explanation and elaboration. Annals of internal medicine Oct.

[29] Hansagi H, Olsson M, Sjoberg S, Tomson Y, Goransson S (2001). Frequent use of the hospital emergency department is indicative of high use of other health care services. Annals of emergency medicine Jun.

[30] Hunt KA, Weber EJ, Showstack JA, Colby DC, Callaham ML (2006). Characteristics of frequent users of emergency departments. Annals of emergency medicine Jul.

[31] Sun BC, Burstin HR, Brennan TA (2003). Predictors and outcomes of frequent emergency department users. Acad Emerg medicine: official J Soc Acad Emerg Med Apr.

[32] Bieler G, Paroz S, Faouzi M (2012). Social and medical vulnerability factors of emergency department frequent users in a universal health insurance system. Acad Emerg medicine: official J Soc Acad Emerg Med Jan.

[33] Soril LJ, Leggett LE, Lorenzetti DL, Noseworthy TW, Clement FM. Characteristics of frequent users of the emergency department in the general adult population: A systematic review of international healthcare systems. *Health policy (Amsterdam, Netherlands)*. May 2016;120(5):452 – 61. 10.1016/j.healthpol.2016.02.006. 10.1016/j.healthpol.2016.02.006 26947060

[34] Dufour I, Chouinard MC, Dubuc N, Beaudin J, Lafontaine S, Hudon C (2019). Factors associated with frequent use of emergency-department services in a geriatric population: a systematic review. BMC Geriatr Jul.

[35] Depelteau A, Racine-Hemmings F, Lagueux E, Hudon C. Chronic pain and frequent use of emergency department: A systematic review. Am J Emerg Med Oct. 2019;14. 10.1016/j.ajem.2019.158492. 10.1016/j.ajem.2019.158492 31706663

[36] Kurteva S, Abrahamowicz M, Weir D, Gomes T, Tamblyn R (2022). Determinants of long-term opioid use in hospitalized patients. PLoS ONE.

[37] Kragh Andersen P, Pohar Perme M, van Houwelingen HC (2021). Analysis of time-to-event for observational studies: Guidance to the use of intensity models. Stat Med Jan.

[38] Lesko CR, Lau B (2017). Bias Due to Confounders for the Exposure-Competing Risk Relationship. Epidemiology.

[39] Lim HJ, Zhang X, Dyck R, Osgood N (2010). Methods of competing risks analysis of end-stage renal disease and mortality among people with diabetes. BMC Med Res Methodol.

[40] Lunn M, McNeil D (1995). Applying Cox regression to competing risks. Biometrics Jun.

[41] Belot A, Abrahamowicz M, Remontet L, Giorgi R (2010). Flexible modeling of competing risks in survival analysis. Stat Med Oct.

[42] Vittinghoff E, McCulloch CE (2007). Relaxing the rule of ten events per variable in logistic and Cox regression. Am J Epidemiol Mar.

[43] Therneau TM, Grambsch PM, Pankratz VS. Penalized Survival Models and Frailty. *Journal of Computational and Graphical Statistics*. 2003/03/01 2003;12(1):156–175. 10.1198/1061860031365.

[44] Suissa S, Dell’Aniello S (2020). Time-related biases in pharmacoepidemiology. Pharmacoepidemiol Drug Saf Sep.

[45] Suissa S, Ernst P. Avoiding immortal time bias in observational studies. Eur Respir J Mar. 2020;55(3). 10.1183/13993003.00138-2020. 10.1183/13993003.00138-2020 32198272

[46] Bykov K, He M, Franklin JM, Garry EM, Seeger JD, Patorno E (2019). Glucose-lowering medications and the risk of cancer: A methodological review of studies based on real-world data. Diabetes Obes Metab Sep.

[47] Hernán MA (2021). Methods of Public Health Research - Strengthening Causal Inference from Observational Data. N Engl J Med Oct.

[48] Giannouchos TV, Kum HC, Foster MJ, Ohsfeldt RL (2019). Characteristics and predictors of adult frequent emergency department users in the United States: A systematic literature review. J evaluation Clin Pract Jun.

[49] Bayoumi I, Dolovich L, Hutchison B, Holbrook A (2014). Medication-related emergency department visits and hospitalizations among older adults. Can family physician Medecin de famille canadien Apr.

[50] Moe J, Camargo CA, Davis RB, Jelinski S, Rowe BH (2019). Frequent emergency department use and mortality in patients with substance and opioid use in Alberta: A population-based retrospective cohort study. Cjem Jul.

[51] Slankamenac K, Zehnder M, Langner TO, Krahenmann K, Keller DI. Recurrent Emergency Department Users: Two Categories with Different Risk Profiles. J Clin Med Mar. 2019;9(3). 10.3390/jcm8030333. 10.3390/jcm8030333 PMC6463097 30857294

[52] Emes M, Smith S, Ward S, Smith A (2019). Improving the patient discharge process: implementing actions derived from a soft systems methodology study. Health Syst (Basingstoke).

[53] Kurian T, Stranges E, Czerlanis C (2021). Standardization of the Discharge Process for Inpatient Hematology and Oncology Using Plan-Do-Study-Act Methodology Improves Follow-Up and Patient Hand-Off. Fed Pract May.

[54] Shah A, Hayes CJ, Martin BC (2017). Characteristics of Initial Prescription Episodes and Likelihood of Long-Term Opioid Use - United States, 2006–2015. *MMWR Morbidity and mortality weekly report*. Mar.

[55] Hadlandsmyth K, Lund BC, Mosher HJ. Associations between initial opioid exposure and the likelihood for long-term use. *J Am Pharm Assoc (*2003*)*. Jan-Feb 2019;59(1):17–22. 10.1016/j.japh.2018.09.005. 10.1016/j.japh.2018.09.005 30409501

[56] Shah A, Hayes CJ, Martin BC (2017). Factors Influencing Long-Term Opioid Use Among Opioid Naive Patients: An Examination of Initial Prescription Characteristics and Pain Etiologies. J Pain Nov.

[57] Dowell D, Haegerich TM, Chou R, CDC Guideline for Prescribing Opioids for Chronic Pain - United States., 2016. *MMWR Recomm Rep*. Mar 18 2016;65(1):1–49. 10.15585/mmwr.rr6501e1. 10.15585/mmwr.rr6501e1 26987082

[58] Calcaterra SLS (2018). Prediction of Future Chronic Opioid Use Among Hospitalized Patients. J Gen Intern Med.

[59] Dasgupta N, Funk MJ, Proescholdbell S, Hirsch A, Ribisl KM, Marshall S. Cohort Study of the Impact of High-Dose Opioid Analgesics on Overdose Mortality. *Pain medicine (Malden, Mass)*. Jan 2016;17(1):85–98. 10.1111/pme.12907. 10.1111/pme.12907 26333030

[60] Paulozzi LJ, Zhang K, Jones CM, Mack KA (2014). Risk of adverse health outcomes with increasing duration and regularity of opioid therapy. J Am Board Family Medicine: JABFM May-Jun.

[61] Paice JA (2019). Managing Pain in Patients and Survivors: Challenges Within the United States Opioid Crisis. J Natl Compr Cancer Network: JNCCN May.

[62] Berry SD, Ngo L, Samelson EJ, Kiel DP (2010). Competing risk of death: an important consideration in studies of older adults. J Am Geriatr Soc Apr.

