# Supplementary material for: Predictors of frequent emergency department visits among hospitalized cancer patients: a comparative cohort study using integrated clinical and administrative data to improve care delivery
Source: BMC Health Serv Res. 2023 Aug 22;23:887. doi: 10.1186/s12913-023-09854-1 (PMC10464437; doi:10.1186/s12913-023-09854-1)
Supplement: Supplementary file 1 — Supplementary Material 1 [file 12913_2023_9854_MOESM1_ESM.docx]

Appendix A. Description of available data on drug, patient, provider and system level characteristics

|  | **Description** | **Measurement** | **Timing of Measurement** | **Functional Form** |
| --- | --- | --- | --- | --- |
| **Opioid-related Characteristics** | | | | |
| *Opioid Dispensations* | | | | |
| ATC code | Anatomical Therapeutic Chemical Classification System code used to identify opioids and other concurrent medications that the patient is taking  Opioids ATC Included: N02A, R05DA | RAMQ prescription claims. | In the community one year prior to admission | N/A |
| Dose | The daily amount of drug taken by patient was calculated based on information about the number of tablets prescribed, strength and number of days’ supply; daily dose was converted to milligram morphine equivalents to facilitate comparison across opioids | From RAMQ prescription claims | The initial post-discharge dispensation | Binary, time-fixed |
| Duration | The days’ supply on the drug claim as entered by the pharmacist | From RAMQ prescription claims | The initial post-discharge dispensation | Binary, time-fixed |
| Type of opioid | Type of opioid ingredient; e.g.: hydromorphone, oxycodone, morphine, fentanyl, etc. | From RAMQ prescription claims | The initial post-discharge dispensation | Categorical, time-fixed |
| Opioid formulation | E.g.: short-acting, long-acting | From RAMQ prescription claims | The initial post-discharge dispensation | Binary, time-fixed |
| Filled within 30 days‘ post-discharge | Patient filled their initial dispensation within 30 days following the index hospitalization | From RAMQ prescription claims | The initial post-discharge dispensation | Binary, time-fixed |
| Filled ≥1 type of opioid | Patient filled more than one type of opioid ingredient on the first dispensation following their index hospitalization | From RAMQ prescription claims | The initial post-discharge dispensation | Binary, time-fixed |
| *Opioid Administration in Hospital* | | | | |
| ATC code | Anatomical Therapeutic Chemical Classification System code used to identify administered opioids | Hospital pharmacy | In hospital | Categorical |
| *Opioid Prescription at Hospital Discharge* | | | | |
| Status of opioid medication | Continued or stopped from community, or newly prescribed at discharge | From patient chart | At hospital discharge | Categorical, time-fixed |
| Presence of a multi-modal pain management regimen | The opioid prescription at hospital discharge as part of multi-modal pain treatment regimen | From patient chart | At hospital discharge | Categorical,  time-fixed |
| **Demographics** |  |  |  |  |
| Age |  | From patient chart | Admission to hospital | Continuous, time-varying |
| Sex | Male, Female | From patient chart | Admission to hospital | Binary, time-fixed |
| Drug insurance status | E.g.: Full copay, partial copay, no copay; serves as proxy for socio-economic status | From RAMQ drug programs | Admission to hospital | Categorical, time-fixed |
| **Co-Existing Illnesses** |  |  |  |  |
| History of mental health conditions | E.g.: Anxiety, depression, psychiatric diagnosis, mood disorder, and post-traumatic stress disorder | ICD-9 from RAMQ medical services and ICD-10 codes from hospitalization data | In community one year prior to admission, in hospital, post-discharge | Binary per condition, time-varying |
| Pain syndromes | E.g.: Chronic back pain, back and neck pain, back disorder, arthritis, migraine, headache, fibromyalgia, fracture | ICD-9 from RAMQ medical services and ICD-10 codes from hospitalization data | In community one year prior to admission, in hospital, post-discharge | Binary per condition, time-varying |
| Health conditions associated with abuse | E.g.: Alcohol abuse, drug abuse | From patient chart. Also from RAMQ medical series and prescription claims | In community one year prior to admission, in hospital, post-discharge | Binary per condition, time-varying |
| Tobacco use | Patient-reported history of tobacco use | From hospital charts | At admission | Binary, time-fixed |
| Cancer diagnosis | E.g.: Metastatic, non-metastatic, lymphoma | ICD-9 from RAMQ medical services and ICD-10 codes from hospitalization data | In community one year prior to admission, in hospital, post-discharge | Binary per condition, time-varying |
| Other comorbidities | E.g.: Acute MI, cerebrovascular diseases, chronic kidney, COPD, diabetes, heart failure, hypertension, ischemic heart disease, liver, obesity | ICD-9 from RAMQ medical services and ICD-10 codes from hospitalization data | In community one year prior to admission, in hospital, post-discharge | Binary per condition, time-varying |
| **Drug and Healthcare utilization** | | | | |
| Use of potential interacting drugs increasing the risk of opioid misuse | E.g.: Selective serotonin reuptake inhibitors, other antidepressants, benzodiazepines, other antipsychotic drugs, central nervous system depressants, psychotropic medication | ATC codes, DIN, Generic Drug name used to extract information from RAMQ prescription claims, hospital data, patient chart. | In community one year prior to admission, in-hospital | Binary per drug, time-varying |
| Use of non-opioid pain medications | E.g.: NSAIDS, COX-2, Acetaminophen, gabapentin, anti-migraine medications, muscle-relaxants, other anti-inflammatories and anti-rheumatoid medications | ATC codes, DIN, Generic Drug name used to extract information from RAMQ prescription claims, hospital data, patient chart. | In community one year prior to admission, in-hospital | Binary per drug, time-varying |
| Receipt of radiotherapy | The patient received radiotherapy | Procedure codes | In community one year prior to admission | Binary, time-fixed |
| Receipt of chemotherapy | The patient received chemotherapy | Procedure codes | In community one year prior to admission | Binary, time-fixed |
| Number of emergency department (ED) visits and hospitalizations | Total number of ED visits and hospitalizations | From RAMQ prescription claims and hospital data | One year prior to hospital admission & one year post-discharge | Categorical, continuous, time-varying |
| Active prescriptions at admission | Total number of active dispensations (duration of the dispensation overlapped with the date of admission) at index hospitalization admission | From RAMQ prescription claims | One year prior to hospital admission | Continuous, categorical, time-fixed |
| *Measures of Care Continuity* | | | | |
| Number of physicians | Number of unique physicians that prescribed an opioid medication to a patient in the year post hospital admission | From RAMQ medical services | One year prior to hospital admission & one year post-discharge | Categorical, continuous, cumulative time-varying |
| Number of dispensing pharmacies | Number of unique pharmacies that a patient has opioid medications dispensed at in the one year post to hospital admission | From RAMQ medical services | One year prior to hospital admission & One year post-discharge | Categorical, continuous, cumulative time-varying |
| **In-hospital Characteristics** | | | | |
| Reason for index hospital admission | Reasons were classified as opioid-related if patient presented to the hospital for cancer-related reasons, respiratory, cardiovascular, etc. | From hospitalization data | During the hospital stay | Binary, time-fixed |
| Type of surgery | For surgical patients only, the type of surgery that the patient received during their index hospitalization | From hospitalization data | During the hospital stay | Categorical, time-fixed |
| Admission to the intensive care unit (ICU) | A record for patient being admitted to the ICU during their index hospitalization | From hospitalization data | From hospitalization data | Binary, time-fixed |
| Length of hospital stay | The duration of the index hospitalizations (days) | From hospitalization data | From hospitalization data | Binary, time-fixed |
| Discharge Destination | Home community, long term care | Patient chart | Upon discharge | Binary, time-fixed |
| RightRx patients | Patients, who were part of the initial randomized controlled trial and electronic medication reconciliation was used | Patient chart | Upon discharge | Binary, time-fixed |
| **Attending Physician Characteristics** | | | | |
| Years of practice | Number of years practiced since graduation from medical school | From hospital chart, Collège des Médecins du Quebec | Upon discharge | Categorical, time-fixed |
| Discharge prescription signed by | Attending physician vs resident | From hospital chart | Upon discharge | Binary, time-fixed |

Appendix B. Codes Used for Drug Classification and Rationale for Opioid Dose Calculations

**Supplement Method B.1.** Inclusion criteria for opioid medications.

ATC codes used to identify opioids: N02A (opioids), R05DA (opium alkaloids and derivatives)

Exclusions: Not all drug forms were included in the analyses. Only patches and tablets of these medications were kept. Injectable, liquid and rectal forms were excluded. Methadone and buprenorphine/naloxone combinations were kept to define subclinical patient populations but were excluded from all dosing/duration calculations as these medications are used to treat addiction and we want to focus on the association of duration/dose of opioids used for pain relief.

**Supplement Method B.2.** Calculation of daily dose and overlapping prescriptions.

The daily dose of each opioid was calculated by first dividing the quantity of units dispensed by the prescription duration to determine the number of units per day, and then multiplying the number of units by the strength. To account for concurrent prescriptions, a subsequent dispensation was considered as an early refill if days of overlap were ≤30% of the previous dispensation duration. Otherwise, the opioids were considered to be taken simultaneously. Daily dose of each dispensation was converted to MME doses using the Center for Disease Control Opioid Morphine Equivalent Conversion Factor and the opioid doses determined to be concurrently dispensed were added together.

Appendix C. Opioid Morphine Equivalent Conversion Factor **^1^**

**Drug Name Conversion Factor**

| Buprenorphine patch^2^ | 12.6 |
| --- | --- |
| Buprenorphine tab or film | 10 |
| Butorphanol | 7 |
| Codeine | 0.15 |
| Dihydrocodeine | 0.25 |
| Fentanyl buccal or SL tablets, or lozenge/troche^3^ | 0.13 |
| Fentanyl film or oral spray^4^ | 0.18 |
| Fentanyl nasal spray^5^ | 0.16 |
| Fentanyl patch^6^ | 7.2 |
| Hydrocodone | 1 |
| Hydromorphone | 4 |
| Levorphanol tartrate | 11 |
| Meperidine hydrochloride | 0.1 |
| Methadone | 3 |
| Morphine | 1 |
| Nalbuphine | 1 |
| Opium | 1 |
| Oxycodone | 1.5 |
| Oxymorphone | 3 |
| Pentazocine | 0.37 |
| Tapentadol | 0.4 |
| Tramadol | 0.1 |

^1^ Centers for Disease Control and Prevention, Atlanta, GA, May 2014.

^2^ The MME conversion factor for buprenorphine patches is based on the assumption that one milligram of parenteral buprenorphine is equivalent to 75 milligrams of oral morphine and that one patch delivers the dispensed micrograms per hour over a 24-hour day. Example: 5 ug/hr buprenorphine patch * 24 hrs = 120 ug/day buprenorphine = 0.12 mg/day buprenorphine = 9 mg/day oral morphine milligram equivalent. In other words, the conversion factor not accounting for days of use would be 9/5 or 1.8. However, since the buprenorphine patch remains in place for 7 days, we have multiplied the conversion factor by 7 (1.8 X 7 = 12.6). In this example, MME/day for four 5 μg/hr buprenorphine patches dispensed for use over 28 days would work out as follows: Example: 5 ug/hr buprenorphine patch * (4 patches/28 days) * 12.6 = 9 MME/day.

^3^ The MME conversion factor for fentanyl buccal tablets, sublingual tablets, and lozenges/troche is 0.13. This conversion factor should be multiplied by the number of micrograms in a given lozenge/troche.

^4^ The MME conversion factor for fentanyl film and oral spray is 0.18. This reflects a 40% greater bioavailability for films compared to lozenges/tablets and 38% greater bioavailability for oral sprays compared to lozenges/tablets.

^5^ The MME conversion factor for fentanyl nasal spray is 0.16, which reflects a 20% greater bioavailability for sprays compared to lozenges/tablets.

6 The MME conversion factor for fentanyl patches is based on the assumption that one milligram

of parenteral fentanyl is equivalent to 100 milligrams of oral morphine and that one patch delivers

the dispensed micrograms per hour over a 24 hour day. Example: 25 ug/hr fentanyl patch * 24 hrs=

600 ug/day fentanyl = 60 mg/day oral morphine milligram equivalent. In other words, the conversion

factor not accounting for days of use would be 60/25 or 2.4. However, sincethe fentanyl patch

remains in place for 3 days, we have multiplied the conversion factor by 3 (2.4 X 3 = 7.2).

In this example, MME/day for ten 25 μg/hr fentanyl patches dispensed for use over 30 days would work

out as follows: Example: 25 ug/hr fentanyl patch * (10 patches/30 days)* 7.2 = 60 MME/day.

*Sources:*

1. Centers for Medicare & Medicaid Services. Opioid Oral Morphine Milligram Equivalent (MME) Conversion Factors. https:\\www.cms.govMedicarePrescription-Drug-CoveragePrescriptionDrugCovContraDownloadsOpioid-Morphine-EQConversion-Factors-vFeb-.pdf. Accessed: September 5, 2019
2. Svendsen, K., Borchgrevink, P., Fredheim, O., Hamunen, K., Mellbye, A., & Dale, O. (2011). Choosing the unit of measurement counts: the use of oral morphine equivalents in studies of opioid consumption is a useful addition to defined daily doses. Palliative Medicine, 25(7), 725–732. <http://doi.org/10.1177/0269216311398300>

Appendix D. Results from assessing the proportional hazards (PH) assumption in the main Cox PH model (global p-value = 0.0021)

| **Covariate** | **P-value** |
| --- | --- |
| Emergency department visits/hospitalizations, ≥1 | **0.040** |
| Ambulatory visits, ≥15 | 0.89 |
| Radiotherapy | 0.31 |
| Chemotherapy | 0.43 |
| Charlson Comorbidity Index, ≥3 | 0.16 |
| History of renal disease | 0.86 |
| History of diabetes | 0.20 |
| History of heart disease | **0.021** |
| History of lung cancer | 0.41 |
| History of opioid use | 0.07 |
| History of benzodiazepine use | 0.90 |
| Cardiac surgery | 0.45 |
| Gastrointestinal surgery | 0.69 |
| Thoracic surgery | 0.93 |
| Unrelated surgery | 0.57 |
| Admission to the ICU | 0.40 |
| Hospital LOS, ≥6 | **0.0091** |
| Discharge prescription of a non-opioid analgesic | 0.67 |

ICU= Intensive care unit; LOS=Length of hospital stay

Statistically significant findings are bolded.

Appendix D.1. Smooth residual plot for the time-dependent effect of history of emergency department (ED) visit in the year proceeding the index hospitalization


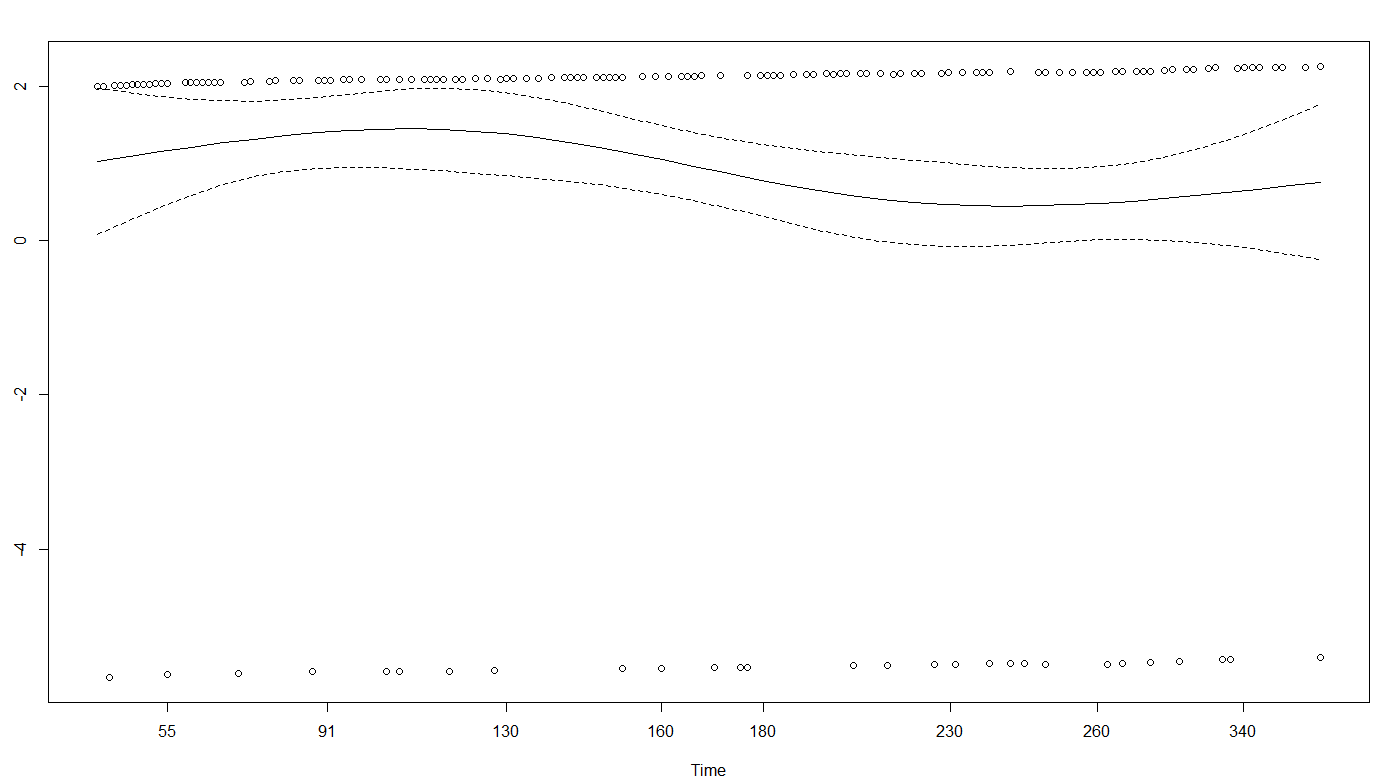


**Supplement Method D.1.** From the graph of the smooth residual plot, we see that history of ≥1 emergency department visits is associated with an increase in risk over the entire follow-up period. This consistent increase in risk is observed under constant proportional hazards effect with a p-value of 0.04 (Appendix D.1) and a HR >1 (HR=1.80). This increase in risk of the outcome associated with an having a history of ≥1 ED visits was even higher during the first three to four months of follow-up.

Appendix D.2. Smooth residual plot for the time-dependent effect of the history of heart disease


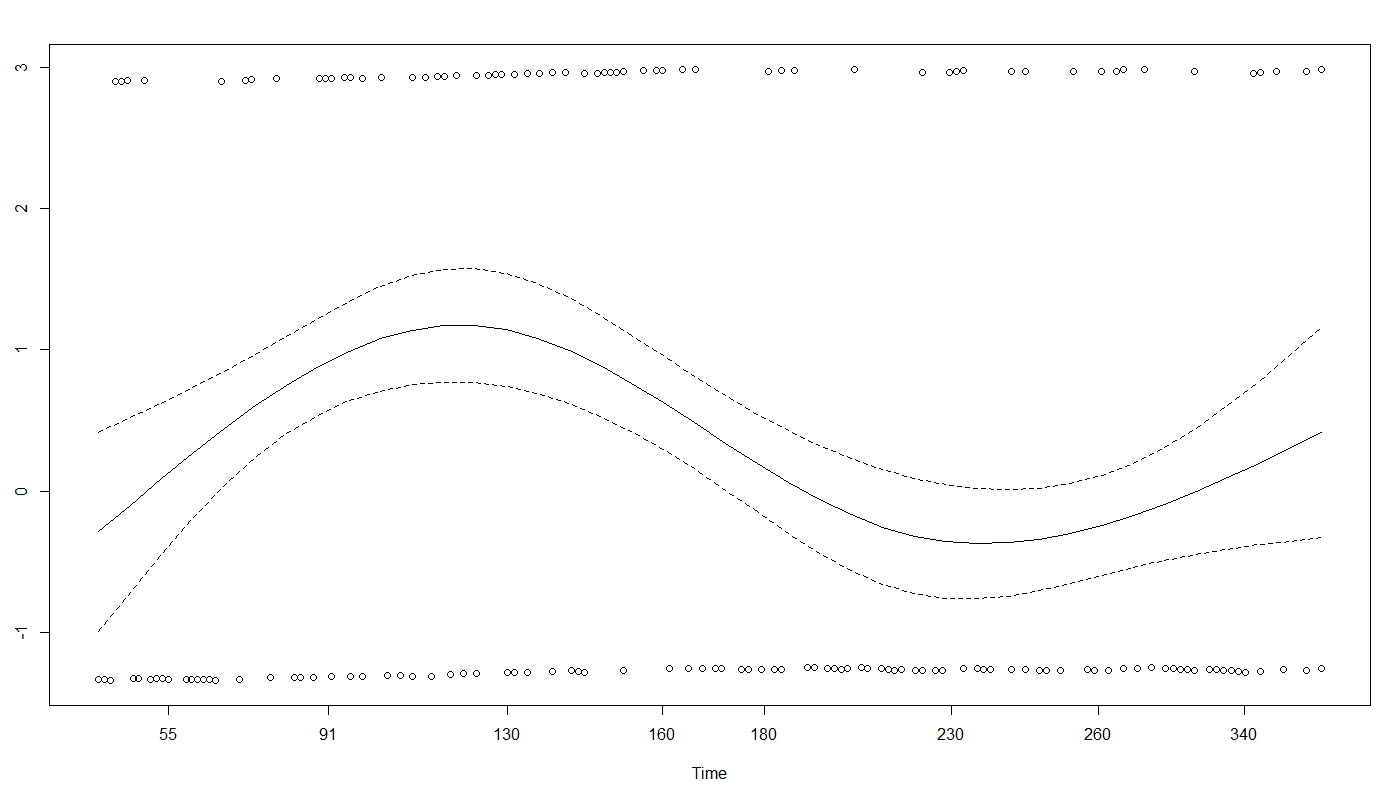


**Supplement Method D.2.** From the graph of the smooth residual plot, the impact of history of heart disease on the hazard increases with time, starting with gradual increases in risk three to four months following hospital discharge to decreasing risk after four months post-discharge. The risk increases again after six months of discharge up until the end of follow-up.

Appendix D.3. Smooth residual plot for the time-dependent effect of the length of hospital stay of the index hospitalization


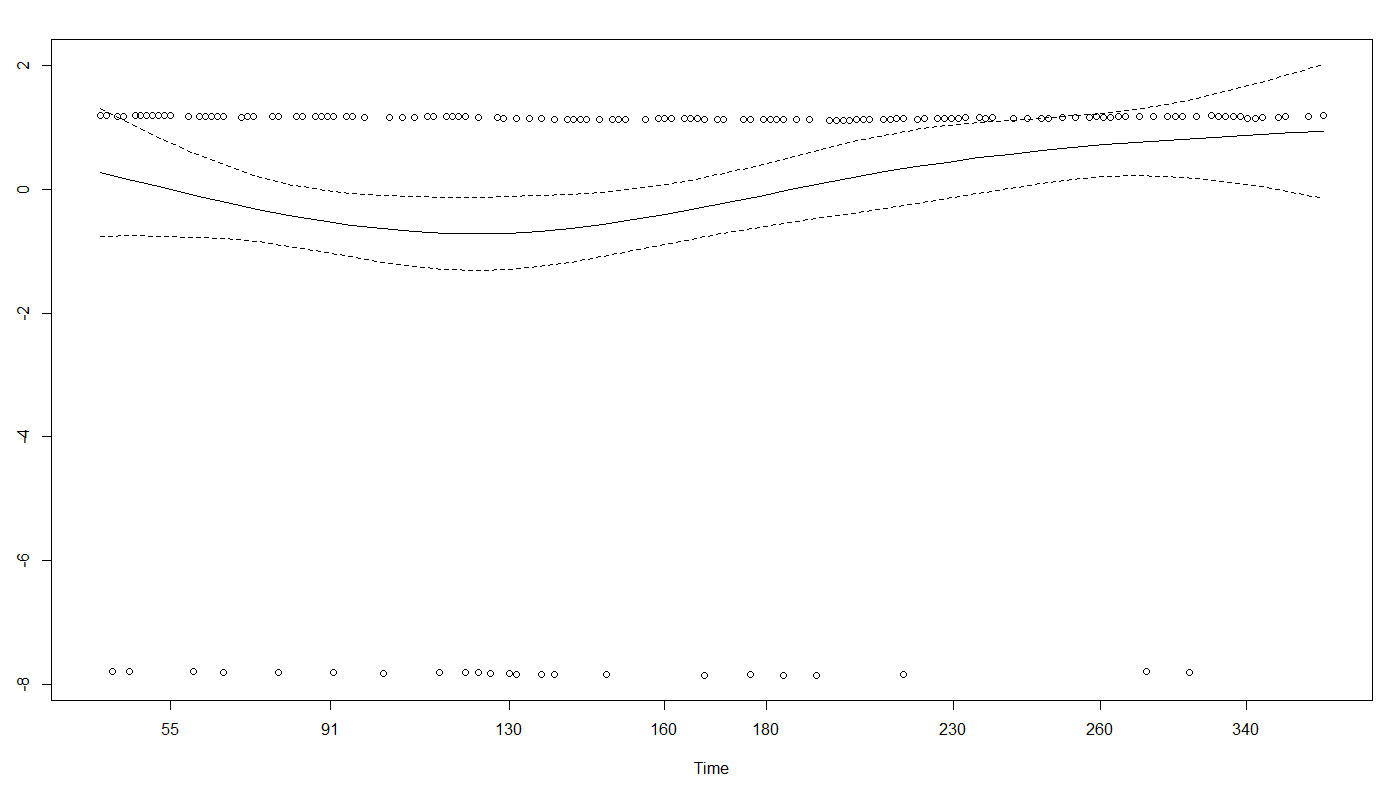


**Supplement Method D.3.** From the graph of the smooth residual plot, we see that having a length of hospital stay of ≥6 days is significant (p-value of 0.0091). The impact of having a length of hospital stay of ≥6 days on the hazard slowly and gradually decreases at first, up until four months of follow-up and then gradually increases with time until the end of follow-up. The crossing hazards could explain why the effect of having a length of hospital stay of ≥6 days on the risk of becoming a frequent ED user is non-significant (HR is close to 1, and the confidential intervals span the null).

Appendix E. Breakdown of the overall reasons for the emergency department visits among the frequent emergency department users as compared to those between 1-3 ED visits in the one-year post-discharge.

| **Reasons, N (%)**^1^ | **Non-frequent ED users (1-3 ED visits), n=472**  **(651 ED visits in total)** | **Frequent ED users (≥4 ED visits), n=182 (728 ED visits in total)** |
| --- | --- | --- |
| Surgical complications (wound complications) | 25 (3.8) | 17 (2.3) |
| Cancer-related | 238 (36.6) | 364 (50.0) |
| Others ^2^ | 154 (23.7) | 292 (44.8) |
| Infections (including post-operative infections, hepatitis, urinary-tract related, tuberculosis) | 180 (27.6) | 312 (42.9) |
| Symptoms involving the cardiovascular system | 175 (26.9) | 321 (44.1) |
| Symptoms involving the digestive system | 111 (17.1) | 181 (24.8) |
| Mental (anxiety, psychotic disorders, depression) | 42 (6.4) | 53 (7.3) |
| Brain conditions (cerebral degenerations, epilepsy) | 18 (2.8) | 12 (1.6) |
| Blood-related (hemorrhage, neutropenia, anemia) | 64 (9.8) | 85 (11.7) |
| Muscle and soft tissue disorders (arthritis, osteoporosis) | 83 (12.7) | 157 (21.6) |
| Respiratory symptoms (COPD, pneumonia) | 130 (20.1) | 193 (26.5) |
| Pain-related (migraine, skeletal and thoracic pain, unspecified pain) | 155 (23.8) | 199 (27.3) |
| Fall/Injuries | 50 (7.7) | 46 (6.3) |
| Follow-up care | 422 (64.8) | 477 (65.5) |

^1^ The overall number does not add up to the number of ED visits as there are usually more than one reasons recorded as part of an ED visit. Percentage is recorded as part of the overall number of ED visits incurred per group: 651 ED visits for patents with 1-3 ED visits, and 728 ED visits for FED users. Patients with no ED visits were excluded from the presentation of the breakdown of the reasons for the ED visits.

^2^ The category ‘other’ includes a combination of multiple reasons for the re-admissions and the ED visits, each of which represented less than 3% of the overall number of events. These reasons included: unspecified screenings, adverse drug events, nausea, fatigue, diabetes, renal failure, eye disorders, anemia, neuropathic disorders, skin disorders, and unspecified disorders.
